# The Texas Pharmaceutical Association

**Published:** 1888-05

**Authors:** 


THE GROSS MEDICAL COLLEGE has recently purchased a new college building, at a cost of $16,000.

---

THE Texas State Pharmaceutical Association, which was to have held its annual meeting in Austin, May 8th, has postponed it till June 12th, prox.

